# Adipokines in Pregnancy: A Systematic Review of Clinical Data

**DOI:** 10.3390/biomedicines11051419

**Published:** 2023-05-11

**Authors:** Noura Kabbani, Matthias Blüher, Holger Stepan, Michael Stumvoll, Thomas Ebert, Anke Tönjes, Susanne Schrey-Petersen

**Affiliations:** 1 Department of Obstetrics, University of Leipzig Medical Center, 04103 Leipzig, Germany; 2 Medical Department III—Endocrinology, Nephrology, Rheumatology, University of Leipzig Medical Center, 04103 Leipzig, Germany; 3 Helmholtz Institute for Metabolic, Obesity and Vascular Research (HI-MAG) of the Helmholtz Zentrum München, The University of Leipzig and University Hospital Leipzig, 04103 Leipzig, Germany

**Keywords:** adipokines, pregnancy, preeclampsia, gestational diabetes, intrauterine growth

## Abstract

Adipokines are signaling proteins involved in metabolic, endocrinological, vascular and immunogenic processes. Associations of various adipokines with not only insulin resistance but also with increased insulin sensitivity, increased systolic blood pressure, and atherosclerosis highlight the significance of adipokines in several components of metabolic syndrome and metabolic diseases in general. As pregnancy presents a unique metabolic state, the role of adipokines in pregnancy, and even in various pregnancy complications, appears to be key to elucidating these metabolic processes. Many studies in recent years have attempted to clarify the role of adipokines in pregnancy and gestational pathologies. In this review, we aim to investigate the changes in maternal adipokine levels in physiological gestation, as well as the association of adipokines with pregnancy pathologies, such as gestational diabetes mellitus (GDM) and preeclampsia (PE). Furthermore, we will analyze the association of adipokines in both maternal serum and cord blood with parameters of intrauterine growth and various pregnancy outcomes.

## 1. Introduction

Pregnancy is a unique state accompanied by various alterations in physiological metabolic, endocrinological, vascular and immunogenic processes. These alterations create an optimal environment for the fetus and allow for appropriate fetal development. While higher insulin secretion and largely stable insulin sensitivity are physiologically associated with the first trimester of pregnancy, metabolic and endocrinological processes during the second and third trimesters are characterized by insulin resistance and, therefore, relatively insufficient insulin secretion. This phenomenon is physiologically countered through hypertrophy or hyperplasia of the pancreatic β-cells and subsequently elevated insulin secretion [1]. When this adaptation proves inadequate, insulin resistance could develop into gestational diabetes.

Adequate intrauterine and placental perfusion are maintained by proper placentation, appropriate development of placental blood vessels and sufficient intrauterine blood supply. This is facilitated in part by a physiological decrease in maternal median blood pressure and an increase in maternal plasma volume. Defective placentation, which is thought to cause placental dysfunction, is associated with both fetal and maternal complications of pregnancy, as it can lead to insufficient fetal blood supply, as well as hypertensive disorders of pregnancy on the maternal side.

Adverse intrauterine conditions can lead to insufficient oxygen and nutrient (among others) supply of the fetus and interfere with intrauterine growth, and therefore to possible intrauterine growth restriction (IUGR). It is, therefore, imperative to closely examine these adapted processes to better understand the pathophysiology behind common pregnancy diseases. In this regard, adipokines present an interesting cross-link between physiological pregnancy adaptations and pathological processes. Particularly of interest is the function of these secretory proteins in metabolic processes throughout pregnancy and the fetal period. While adipokines such as leptin and adiponectin have been intensively researched over the last twenty years, novel adipokines such as progranulin and neuregulin have only recently emerged as possible biomarkers in pregnancy and the perinatal period. In this review, we aim to investigate the changes in maternal adipokine levels in physiological gestation, as well as the association of adipokines with pregnancy pathologies such as gestational diabetes mellitus (GDM) and preeclampsia (PE). For this purpose, we will examine eleven novel adipokines that have been the subject of such studies since 2010. The adipokines considered in this review include resistin, chemerin, retinol-binding-protein 4 (RBP4), growth differentiation factor 15 (GDF15), irisin, adipocyte fatty-acid-binding protein (AFABP), omentin-1, lipocalin-2, visfatin, neuregulin 4 (NRG4) and progranulin. We will focus on their role in physiological pregnancy, pregnancy complications and pregnancy outcomes.

### 1.1. Adipokines

Ever since J.M. Friedman’s laboratory discovered leptin in 1994, the role of adipokines in various metabolic, vascular and immunogenic processes has been recognized. Adipokines are a vast group of signaling proteins and mediators secreted primarily from adipose tissue [2]. Pro-inflammatory adipokines such as leptin correlate positively with the amount of adipose tissue and obesity, suggesting an accompanying chronic, low-grade inflammation [3]. In an analogous manner, anti-inflammatory adipokines such as adiponectin, though fewer, have been found to be decreased in overweight individuals [2]. Associations of various adipokines with insulin resistance, but also with increased insulin sensitivity, increased systolic blood pressure, and atherosclerosis, highlight the significance of adipokines in several components of metabolic syndrome and metabolic diseases in general. As pregnancy presents a unique metabolic state, the role of adipokines in pregnancy and even in various pregnancy complications appears to be key to clarifying these metabolic processes.

### 1.2. Gestational Diabetes Mellitus

Gestational diabetes mellitus (GDM) is a metabolic disorder that affects approximately 2–10% of pregnancies in the USA and Europe, and is characterized by glucose intolerance and maternal hyperglycemia with initial onset during pregnancy [4]. During the beginning of pregnancy, insulin secretion is physiologically elevated in the pancreatic β-cells, and this period can even be associated with states of hypoglycemia. However, usually around the beginning of the second trimester, insulin resistance rises while insulin secretion remains unchanged, leading to maternal hyperglycemia. When the pancreatic β-cells fail to compensate, GDM results. The majority of women diagnosed with GDM return to a normoglycemic state after pregnancy; however, studies show that 17–50% will later develop some form of glucose intolerance later in life [4]. GDM also comes with a slew of complications for both mother and child, including atherosclerosis in GDM mothers and hypoglycemia, as well as large for gestational age (LGA) birthweight in GDM offspring.

### 1.3. Preeclampsia

Preeclampsia (PE) is a serious pregnancy condition associated with elevated blood pressure, which is first diagnosed during pregnancy, proteinuria, edema and other signs of organ dysfunction. While the exact pathophysiology of preeclampsia is still the subject of current research, it is generally accepted that the root of the problem starts with faulty placentation and defective placental blood vessel development. This is often accompanied by a general endothelial dysfunction, which can affect multiple organ systems, and can overall lead to an insufficient blood supply of the placenta and, in turn, the fetus. Preeclampsia and other hypertensive gestational disorders are also associated with higher mortality for both mother and infant. Since the only causal therapy is premature delivery of the baby, research regarding prevention and early diagnosis is key.

### 1.4. Intrauterine Growth and Fetal Programming

“The fetal origin of adult disease” was first coined by David Barker in 1990 [5]. His theory of fetal programming stipulates that the intrauterine environment of a fetus not only affects the fetus itself, but also leads to an adaptation of metabolic fetal processes that can later shape the health trajectory in childhood and adulthood. This adaptation ensures the optimal survival of the fetus in the given intrauterine environment during pregnancy and later after birth. In the case of pathophysiological pregnancy states, however, an altered fetal environment results, and the consequent fetal adaptations are unsuitable for the environment after birth. To this end, Barker noted a higher rate of cardiovascular events in adults who had lower birth weights [5]. Therefore, the metabolic processes during pregnancy are proven to have a lasting effect on the fetus, not only in pregnancy but long after birth and even much later in adulthood.

A key common denominator of abnormal birthweight, PE and GDM is the heightened risk of prospective cardiovascular and metabolic diseases, including metabolic syndrome in offspring [6,7,8]. Consequences of this disease, such as atherosclerosis and diabetes, can result in heart disease or even stroke. Mohseni et al., suggest a U-shaped correlation between birthweight and cardiovascular disease, with both low (<2000 g) and very high (>4000 g) birthweight considered as risk factors [8]. This illustrates the importance of researching in detail the regulatory function of novel adipokines in the perinatal period.

## 2. Materials and Methods

We searched PubMed/MEDLINE databases for published studies considering the adipokines resistin, chemerin, RBP4, GDF15, irisin, AFABP, omentin-1, lipocalin-2, visfatin, NRG4 and progranulin in gestational diabetes from 2013 through February of 2023, and, due to their smaller number, studies considering the aforementioned adipokines and preeclampsia or intrauterine growth from 2010 through February of 2023. We excluded studies that either assessed women with all types of diabetes and not GDM specifically, studies based on mRNA expression, in vitro or cell culture studies, editorials and comments, animal model studies, studies that considered adipokine levels in materials other than blood, serum or plasma, and studies which failed to report the number of cases and/or controls. Of the 133 articles yielded with this search, 122 studies relating to adipokines and pregnancy met our criteria and were included in this review. We also included a summary of all the studies that we reference throughout this article in the form of a table (see Supplementary Materials).

## 3. Literature Review

### 3.1. Resistin

Resistin is an insulin resistance-inducing adipokine. It has been found to play a role in the development of GDM [9], is expressed in various organs where insulin acts, and seems to be upregulated in obesity. Studies have shown that resistin is secreted not only by adipocytes but also by monocytes and macrophages. A proposed explanation for the glucose tolerance impairing properties of resistin in animal models includes its interference with hepatic glucose uptake [10]. Nevertheless, the role of resistin in physiological pregnancy and its possible associations with gestational pathologies is still being researched. Several studies looking at resistin in pregnancy have been published since 2010.

#### 3.1.1. Resistin in Physiological Pregnancy

There are conflicting reports on the dynamic of resistin levels in physiological pregnancy. A multicenter prospective study on 232 Saudi women showed that resistin levels decreased significantly from the first to the second trimester in healthy pregnant women [11]. The overall dynamic of resistin levels throughout pregnancy was, however, not described [11]. On the other hand, a prospective cohort study including 91 pregnant women found that resistin levels were significantly elevated in the second trimester and remained elevated throughout gestation [12]. A randomized controlled trial of 123 participants supported these findings [13]; however, the control group here comprised women who had a history of GDM in previous pregnancies, although they displayed normal glucose tolerance during the study. A further review speculated that, based on the findings of a previous study [14], increased resistin levels in the third trimester could lead to higher insulin resistance and postprandial hyperglycemia [15].

#### 3.1.2. Resistin in GDM and PE

Numerous studies have analyzed resistin levels in GDM pregnancies. Five studies, including two case-control studies, two cohort studies and a prospective observational study with 133, 80, 236, 480 and 196 participants, respectively, as well as one meta-analysis including 11 studies, found no significant difference in resistin levels between GDM and non-GDM pregnant controls [16,17,18,19,20,21]. Four further studies, including two case-control studies with 90, 208, 140 and 130 participants, respectively, observed resistin to be significantly elevated in GDM patients [22,23,24,25], and maternal serum resistin levels were found to correlate positively with the known GDM risk factor LDL cholesterol [22]. Resistin levels in the first and second trimesters were also reported to be positively associated with glycated hemoglobin (HbA1c) in GDM mothers [25]. A cohort study including 80 pregnant women also found maternal resistin levels in pregnant women with GDM to be positively correlated with intestinal fatty-acid-binding protein (I FABP) levels [26]. Furthermore, a systematic review of pregnant women with GDM concluded that maternal resistin levels overall increase with progression of the gestation and finally reach a saturation level [9]. On the other hand, a case-control study comprising 73 pregnant women found that serum resistin levels of GDM patients were significantly lower at 31 weeks gestation in comparison to healthy controls [27], while a prospective observational trial of 75 GDM pregnancies observed an association between low resistin levels and poorly controlled GDM [28].

The relationship between resistin concentrations and PE has also been tackled in recent years. Here, the results seem to be slightly more homogenous. Five studies with 91, 196, 40, 153 and 60 participants, respectively, reported increased resistin serum levels in PE pregnancies in comparison to healthy pregnant controls [12,19,29,30,31]. A cohort study also examined placental resistin expression to account for this increase in resistin levels, and due to similar findings between PE and non-PE patients, suggested that the elevated resistin levels may not be of placental origin [29]. In accordance with these findings, a decrease in resistin levels four weeks postpartum in women with PE in comparison to pregnant women without PE was demonstrated in an observational cohort study [31]. Conversely, one case-control study with 117 pregnant women found no significant difference in maternal serum resistin levels between PE patients and healthy controls; however, it reported a positive correlation between serum resistin and subcutaneous fat area in PE women [32]. A secondary analysis of 64 patients who had a history of PE and healthy controls 10 years after delivery found maternal resistin concentrations to be similar among both groups [33].

#### 3.1.3. Resistin and Parameters of Intrauterine Growth

In regard to parameters of intrauterine growth, one case-control study with 153 participants found maternal serum resistin levels to correlate negatively with child weight at birth; however, no association was found between maternal resistin levels and maternal BMI [30]. In addition, no significant differences were found between resistin levels in maternal serum and pregnancy outcomes such as LGA, small for gestational age (SGA) births and healthy controls in a cohort study [19].

#### 3.1.4. Cord Blood Resistin

The analysis of resistin levels in cord blood at birth also yielded several interesting results. A case-control study with 73 participants found cord resistin levels to be significantly lower in the offspring of mothers who had GDM in comparison to healthy controls [27]. Higher levels of cord resistin levels were observed in a cohort study in infants with macrosomia at birth when compared to normal-weight newborns [23].

Resistin appears to play an important role in GDM and possibly the pathogenesis of impaired glucose tolerance, and increased maternal resistin levels could be associated with the presence of maternal PE. Further studies are required in order to establish the possibility of using resistin as a biomarker for various pregnancy outcomes.

### 3.2. Chemerin

Chemerin is also known as retinoic acid receptor responder protein 2 (RARRES 2). It is mainly expressed in adipocytes, hepatocytes and pneumocytes. The pro-inflammatory properties of chemerin stem from its stimulation of the chemotaxis of macrophages and dendritic cells. Chemerin has also been found to have insulin resistance-inducing properties and to correlate positively with BMI and HOMA-IR [34].

#### 3.2.1. Chemerin in Physiological Pregnancy

Three separate studies, including two cross-sectional cohort studies with 222 and 212 women, respectively, as well as one case-control study with a total of 17 subjects, reported elevated chemerin levels in the serum of healthy mothers during pregnancy [35,36,37]. In one case-control study, it was speculated that this increase might help in countering pregnancy-induced insulin resistance and improve impaired glucose tolerance [37]. This study also concluded that the increased chemerin levels later decreased significantly post-partum in healthy pregnant subjects [37]. However, not all studies supported this trend—a randomized controlled trial study with a total of 123 subjects, all with higher GDM risk, found that circulating chemerin levels in pregnant mothers did not change significantly throughout gestation [13].

#### 3.2.2. Chemerin in GDM and PE

Numerous studies have looked at chemerin levels in maternal serum in cases of GDM. The results are very heterogeneous; five studies, including two meta-analyses, did not find a significant difference in chemerin levels between GDM mothers and healthy pregnant controls [13,35,38,39,40], while one of these studies suggested that maternal chemerin levels were higher in the case of maternal obesity [38]. Ebert et al., propose that elevated chemerin levels are associated with pregnancy status and not with the presence of GDM during gestation [35]. In a cohort study, Fatima et al., found a seven-fold increase in maternal chemerin concentrations in comparison with healthy pregnant controls [41]. Four further cohort studies also supported the finding of elevated chemerin levels during GDM [34,42,43,44], with a focus on increased chemerin levels in early and mid-pregnancy [42,43]. A nested case-control study also supported this finding [45]. Two cohort studies likewise established a positive correlation between chemerin and obesity in GDM patients [34,44]. On the contrary, in a cohort study, Yang et al., reported lower serum chemerin levels in the first trimester in patients with GDM when compared to healthy controls; however, chemerin levels were later significantly elevated in the third trimester [36]. This result was also observed in two case-control studies, where lower chemerin was associated with a higher risk for GDM [37,46].

With regard to PE, reports were much more uniform. Two case-control studies, two cohort studies, one cross-sectional study, as well as one systemic review found chemerin levels in maternal serum to be significantly elevated in patients with PE [47,48,49,50,51,52]. Stepan et al., demonstrated that chemerin levels are not only increased during preeclamptic pregnancies but also remain elevated 6 months post-partum [47]. Several studies also reported a correlation of chemerin levels with the severity of PE [48,49,51]. A positive association between chemerin levels towards the end of pregnancy in PE patients and maternal hypertension after birth was also described by Chen et al., in a prospective cohort study [50].

#### 3.2.3. Chemerin and Parameters of Intrauterine Growth

Elevated chemerin levels in maternal serum were not only associated with GDM and PE pregnancies, but were also positively associated with fetal weight [41]. Cetin et al., also reported a negative correlation between chemerin levels in maternal serum at birth and various neonatal outcomes such as birthweight, the APGAR score at 1 and 5 min, as well as the gestational week at delivery [49].

#### 3.2.4. Cord Blood Chemerin

In an observational study by Van Poppel et al., chemerin concentrations in arterial cord blood were elevated in the offspring of GDM mothers, while concentrations in venous cord blood were elevated in the offspring of obese mothers [38]. Through multivariate analyses, the authors elucidated that cord chemerin was not associated with birthweight but correlated instead with maternal chemerin concentrations at birth [38].

In summary, chemerin appears to be significantly increased in PE, while changes in chemerin levels in patients with GDM vary between studies. The association of both maternal and cord chemerin with birthweight also remains unclear; further studies are necessary in order to pinpoint the role of chemerin in intrauterine growth.

### 3.3. RBP4

RBP4 is a known member of the lipocalin family and is expressed mainly by adipocytes and hepatocytes. While RBP4 appears to be independent of BMI, it has been associated with obesity and insulin resistance [53].

#### 3.3.1. RBP4 in GDM and PE

While changes in RBP 4 levels during physiological pregnancy have not yet been investigated, several studies examining its levels in gestational pathologies such as GDM and PE have been published in recent years. Two cohort studies found increased levels of RBP4 in maternal serum in GDM pregnancies in comparison to normal healthy gestations [54,55], and identified RBP4 as a risk factor for the development of GDM, especially among women of a higher age group [55]. A further observational study supported these findings and found elevated RBP4 levels in maternal serum in early pregnancy to be associated with GDM risk [56]. These results were also maintained by two meta-analyses [57,58]. Conversely, three cohort studies found no statistical difference in RBP4 levels between GDM mothers and healthy pregnant subjects [34,59,60]. A cross-sectional study investigating RBP4 levels in the early second trimester in GDM pregnancies also reached a similar conclusion [61]. In a systemic review, RBP4 was found to be possibly altered in pregnancy; however, it did not seem to be predictive of GDM [62].

In studies concerning PE, results were varying. One case-control study found significantly lower maternal RBP4 levels in PE pregnancies compared to healthy gestations [63]. A retrospective case-control study investigating RBP4 levels in the first trimester during early onset PE reported them to be significantly elevated when compared to normal pregnancies [64]. This was consistent with the findings of Mendola et al., who observed this association to be true of preterm but not of term PE [65]. Two further studies reported elevated RBP4 levels in PE [66,67], whereas five studies found no significant differences in RBP4 levels between PE and non-PE subjects [59,68,69,70,71]. Nonetheless, Masuyama et al., described circulating maternal RBP4 to be significantly increased in overweight individuals with late-onset PE in comparison to overweight individuals with normotensive gestations and normal-weight individuals with late-onset PE [71]. Further, this finding was not true of normal and overweight individuals with early onset PE. Seeing as hyperinsulinemia may be directly associated with hypertension and resulting endothelial dysfunction, the authors speculate that RBP4 could perhaps play a role in the pathophysiology of late-onset PE by inducing insulin resistance in obese women [71].

#### 3.3.2. RBP4 and Parameters of Intrauterine Growth

Maternal RBP4 levels might also be associated with various parameters of intrauterine growth. Fruscalzo et al., reported lower RBP4 levels in mothers who later developed fetal growth restriction (FGR) [68], and found decreased RBP4 levels to correlate with a birthweight < 3% in SGA infants, as well as delivery after 37 weeks of gestation [72]. Mendola et al., reported elevated RBP4 throughout gestation in pregnancies that resulted in preterm birth [65]. Nevertheless, a cross-sectional study did not find maternal RBP4 levels to vary significantly between SGA neonates and normal pregnancies [66]. Similar findings were reported by a further cohort study, where maternal serum RBP4 was observed to be similar in women who later had SGA or LGA neonates [59].

#### 3.3.3. Cord Blood RBP4

RBP4 levels in cord blood have also been investigated. A nested case-control study found RBP4 to be elevated in the female offspring of GDM mothers but not in male offspring [73], while another case-control study reported lower cord levels of RBP4 in PE offspring [63]. A further case-control study found cord RBP4 to be elevated in LGA in comparison to appropriate for gestational age (AGA) infants and to be positively correlated with birthweight z score [74]. Yang et al., also propose a 0.28 increase in birth weight z score for each standard deviation (SD) increment in cord blood RBP4 [74].

While findings concerning maternal RBP4 in GDM and PE seem to vary, it is suggested that RBP4 could possibly be involved in the pathophysiology of late-onset PE by stimulating insulin resistance in obese mothers. Furthermore, cord RBP4 appears to be increased in GDM offspring and decreased in PE offspring.

### 3.4. GDF15

GDF15 was first identified as macrophage-inhibiting cytokine 1 (MIC-1) and is a member of the transforming growth factor beta superfamily [75]. GDF15 appears to regulate inflammatory pathways and is involved in various processes such as angiogenesis, cell repair and apoptosis [75]. While GDF15 is expressed in several organs, it is highly expressed in the placenta.

#### 3.4.1. GDF15 in Physiological Pregnancy

In a longitudinal study examining GDF15 levels in all three trimesters in healthy pregnancies, GDF15 was found to increase significantly with gestational age and reach its highest level in the third trimester [76]. This trend was also reported in a case-control study [77].

#### 3.4.2. GDF15 in GDM and PE

Only one study concerning GDF15 levels in maternal serum and GDM has been published thus far. However, a case-control study with 40 pregnant patients with GDM and 40 healthy pregnant controls found significantly higher levels of GDF15 in the serum of GDM patients during the third trimester. Furthermore, it demonstrated through a logistic regression analysis an increasing risk of GDM diseases with increasing maternal GDF15 levels [78]. Nevertheless, no association was found between GDF15 levels and various perinatal outcomes [78].

In contrast, quite a few reports on the association of GDF15 with PE have emerged in recent years, although they provide varying results. A case-control study found significantly reduced levels of GDF15 in maternal serum in the third trimester in PE patients when compared to healthy pregnant subjects [76]. This reduction was especially noticeable in the case of late-onset PE, while GDF15 levels in the first trimester were similar in both PE and non-PE groups [76]. Other studies did not, however, support these findings; Cruickshank et al., reported higher GDF15 levels in PE patients in multiple cohorts, with GDF15 elevation also observed in women who were more likely to develop PE [79]. Similarly, a cross-sectional study also observed higher median GDF15 levels in women with PE in comparison to healthy pregnant controls, and this elevation was most profound in early-onset PE [80]. Yuksel at al. propose this increase to be a result of cytokine-induced endothelial injury, which is involved in the pathogenesis of PE. Conversely, a case-control study reported no difference in circulating GDF15 levels at 19–24 or 25–37 weeks of gestation between patients with early-onset or late-onset PE and normotensive pregnancies [77]. However, in cases of preterm PE, significantly increased GDF15 levels at 30–34 weeks were observed, although this association was not true for patients who later developed PE [77].

#### 3.4.3. Cord Blood GDF15

The associations of GDF15 levels in cord blood with various parameters of intrauterine growth were elucidated in a cohort study. Here, significantly higher levels of GDF15 were described at birth in comparison to subsequent time points in both appropriate for gestational age (AGA) and SGA infants [81]. While they were similar in both SGA and AGA infants, GDF15 levels decreased at around the age of 4 months and continued to decline until 2 years of age, where they reached near adult levels, but this decrease was more profound in SGA infants [81].

In summary, the findings support an increase in maternal GDF15 in GDM, while they remain varied in the case of PE. Further studies are needed to assess the role of GDF15 with parameters of intrauterine growth.

### 3.5. Irisin

First discovered in 2002, irisin results through the cleavage of its precursor, fibronectin type III domain-containing protein 5. Due to its production involving muscular contraction, it has also been classified as a myokine [82]. In addition, it plays a role in the conversion of white adipose tissue to brown adipose tissue and has been associated with processes involved in diabetes and hypertension.

#### 3.5.1. Irisin in Physiological Pregnancy

While there were no findings concerning irisin levels in normal pregnancy, a prospective cohort study reported higher serum levels of the irisin precursor fibronectin type III domain-containing protein 5 in pregnant women during the entire gestation when compared with non-pregnant healthy controls [83].

#### 3.5.2. Irisin in GDM and PE

Three studies also investigated irisin levels in GDM patients. One case-control study with 74 GDM mothers and 74 matched healthy controls found significantly higher irisin levels in GDM patients after delivery; however, irisin levels were similar between both groups during pregnancy [84]. Similarly, a cohort study with 28 mother/newborn pairs found no difference in maternal irisin levels between diabetic and non-diabetic mothers, but patients with other forms of diabetes other than GDM were also included in the study [85]. In contrast to these findings, a case-control study including 130 GDM patients and 140 BMI-matched healthy pregnant controls found significantly decreased maternal irisin levels in GDM mothers when compared to controls [86]. The authors report that this difference in concentration was no longer present three months after birth [86].

The association of irisin with PE was examined in a prospective cohort study. Garcés et al., reported no changes in maternal serum irisin levels during pregnancy in women with PE, but noted that maternal irisin levels decreased significantly in PE women during the third trimester in comparison to healthy pregnant controls [83].

#### 3.5.3. Irisin and Parameters of Intrauterine Growth

In order to explore the association of maternal serum irisin levels with intrauterine growth, Ökdemir et al., conducted a cohort study including 84 mothers and neonates who were classified according to birthweight [87]. They found similar levels of maternal irisin in mothers with SGA, appropriate for gestational age (AGA) and LGA births, and observed a negative correlation of maternal irisin levels with infant anthropometric measurements such as infant neck, chest and arm circumference in AGA infants and normal weight mothers [87]. This correlation disappeared in mothers with excessive gestational weight gain, while maternal irisin levels even correlated positively with infant anthropometric measurements such as arm circumference in SGA infants [87]. The authors concluded that irisin levels might influence infant adiposity in AGA infants but not in neonates who deviate from the norm [87].

#### 3.5.4. Cord Blood Irisin

Infant irisin levels correlated negatively with infant neck circumference, and there was a strong linear correlation between maternal and infant irisin levels [87]. Hernandez-Trejo et al., reported significantly decreased infant irisin levels when compared to maternal serum irisin, but pointed out that this difference was only present in the offspring of mothers who did not have labor before delivery per C-section [85]. The authors did not find any associations between infant irisin levels and the presence of GDM during pregnancy or with maternal BMI at the end of pregnancy [85].

In summary, maternal irisin seems to be decreased in PE, while reports are conflicting in the case of GDM. No association was found between maternal irisin levels and higher birthweight. Additional studies are needed to evaluate the relationship between irisin levels and parameters of intrauterine growth.

### 3.6. AFABP

Since it was first described in 1972, twelve different isoforms of AFABP have been identified. AFABP is a member of the fatty acid-binding protein superfamily and is secreted mainly by adipocytes and macrophages. It has been associated with diabetes, hypertension and cardiovascular events, as well as obesity [88].

#### 3.6.1. AFABP in Physiological Pregnancy

The role of AFABP in physiological pregnancy was described in a systemic review, where AFABP was found to be expressed in the human placenta, and an increase in placental AFABP expression resulted from exposure to a hypoxic environment [15]. It was also suggested that, in cases of hypoxic stress, this protein might be involved in the uptake of lipids [15].

#### 3.6.2. AFABP in GDM and PE

In GDM, there were varying reports. Two studies found no significant differences in maternal AFABP levels between GDM and normoglycemic pregnancies [13,26]; however, the control group in the former comprised women with previous GDM and, therefore, higher GDM risk. A systemic review proposed that AFABP might be predictive of GDM [62], while another reported that maternal AFABP was significantly elevated in GDM mothers in comparison to controls [89]. A third review suggested elevated maternal AFABP levels in GDM patients persist even after birth and lead to a higher risk for the development of type 2 diabetes, as well as metabolic syndrome [90]. Two cohort studies also observed increased levels of maternal AFABP in GDM patients and an association of AFABP with higher GDM risk [42,54]. These findings were in line with the results of a case-control study, which found higher levels of AFABP in maternal serum associated with a 3.7-fold higher risk of GDM [46]. A meta-analysis also concluded that circulating AFABP levels in GDM mothers were higher than in controls [91].

Only two studies were available concerning maternal AFABP levels in PE. In a systematic review, Daskalakis et al., reported an association between increased serum AFABP in the first and third trimester of PE patients [52]. Serum AFABP was also significantly higher in the PE group when compared to controls in a cohort study [92]. Lin et al., proposed that this increase results in disorders of glucose and lipid metabolism, thus increasing insulin resistance and eventually leading to the development of preeclampsia [92].

#### 3.6.3. AFABP and Parameters of Intrauterine Growth

A cross-sectional study also reported that maternal AFABP collected between the 12th and 14th gestational week was positively correlated with birth weight, and that AFABP seemed to be a promising prognostic marker for detecting macrosomia in pregnancy [93].

#### 3.6.4. Cord Blood AFABP

The associations of cord blood AFABP have also been investigated. Joung et al., found AFABP levels in cord blood to be higher in neonates than those found in adults, and that preterm infants showed higher levels of AFABP than full-term infants [94]. They also reported a negative correlation between cord AFABP and gestational age, and lower AFABP levels in SGA infants when compared with AGA and LGA infants [94]. In line with these findings, Ron et al., also reported higher AFABP levels in fetal circulation when compared with AFABP levels in maternal serum and an inverse correlation between cord AFABP and blood glucose of neonates, with a ten-fold increase of umbilical AFABP levels in infants with hypoglycemia [95]. A nested case-control study described a positive correlation between GDM and cord blood AFABP in male offspring, but this correlation was not true for female neonates [96].

Most findings support increased maternal AFABP levels in GDM and PE. Higher maternal AFABP levels are also associated with a higher birthweight, while further studies are needed to assess the role of cord AFABP with pregnancy outcomes and intrauterine growth.

### 3.7. Omentin-1

Omentin-1 is largely expressed in adipose tissue, as well as to a smaller degree in mesothelial and vascular cells. It has been found to have both anti-inflammatory and anti-apoptotic properties and appears to be involved in endothelial dysfunction [97]. The role of omentin-1 in metabolic diseases has been researched often in recent years.

#### 3.7.1. Omentin-1 in Physiological Pregnancy

A few studies have considered maternal serum omentin-1 in physiological pregnancy. A cohort study including 36 pregnant women and 37 healthy, non-pregnant women reported significantly decreased maternal serum omentin-1 levels in uncomplicated pregnant women when compared to the levels found in non-pregnant controls [98]. In line with these findings, a prospective case-control study assessing maternal serum omentin-1 levels at 12–15 weeks gestation found a decrease throughout pregnancy [99].

#### 3.7.2. Omentin-1 in GDM and PE

In the prospective case-control study described above, a stronger decrease in maternal omentin-1 levels throughout pregnancy was observed in women with GDM when compared to healthy pregnant subjects [99]. Similarly, Mierzyński et al., reported significantly decreased maternal serum omentin-1 concentrations in GDM mothers when compared to healthy pregnant controls [100]. This trend was also observed by Abell et al., who described in a randomized trial lower maternal omentin-1 levels at 12 to 15 weeks gestation in women who developed GDM when compared to healthy pregnant subjects, and reported that despite this decrease, omentin-1 did not improve the ability to distinguish GDM patients from non-GDM pregnancies significantly [101,102]. Likewise, a prospective cohort study also reported lower maternal serum omentin-1 in subjects with GDM [103]. This conclusion was consistent with the findings of two meta-analyses [40,104]. In a cohort study, Papatheodorou et al., reported a negative correlation between omentin-1 in maternal serum and insulin; however, this association was significant only between non-overweight/obese subjects and not between overweight/obese subjects [105]. A systematic review proposed omentin-1 to be useful in predicting cardiovascular injuries associated with GDM [106].

In comparison, fewer studies were available when evaluating the relationship between serum omentin-1 and PE. A cross-sectional study reported significantly lower maternal serum omentin-1 levels in women with PE when compared to healthy controls, and reported that in PE patients, serum omentin-1 correlated with BMI as well as systolic blood pressure [107]. The authors also reported an association between serum omentin-1 levels and the severity of PE, with lower levels found in cases of severe PE [107].

#### 3.7.3. Omentin-1 and Parameters of Intrauterine Growth

A case-control study assessing maternal serum omentin-1 levels in 40 mothers with either IUGR neonates or AGA neonates did not find any significant differences in maternal omentin-1 levels in AGA or IUGR neonates [108]. In addition, a cohort study assessing omentin levels in women with GDM who delivered at term, experienced symptoms of threatened preterm labor, or delivered spontaneously preterm and healthy pregnant women, found no significant differences in maternal omentin-1 concentrations between these cohorts [100]. However, the authors reported a significant correlation between maternal serum omentin-1 concentrations and the incidence of preterm birth [100].

#### 3.7.4. Cord Blood Omentin-1

In the case-control study mentioned above, cord omentin-1 levels were similar between AGA and IUGR infants [108]. Furthermore, cord serum omentin-1 was significantly higher than levels found in maternal serum [108]. The authors suggest that this elevation in fetal omentin-1 levels reflects an enhanced growth promotion [108]. A secondary analysis of the cord blood of term neonates grouped based on their birthweight in SGA, AGA and LGA yielded increased omentin-1 levels in SGA neonates when compared to AGA and LGA neonates, and slightly lower omentin-1 levels in LGA neonates [109]. In their prospective cohort study, Franz et al., reported lower omentin-1 levels in the offspring of GDM mothers when compared to the offspring of uncomplicated pregnancies [99].

In summary, maternal omentin-1 levels appear to be decreased in both PE and GDM. However, findings concerning omentin-1 levels in maternal and cord blood and parameters of intrauterine growth, as well as pregnancy outcomes, were varied, and further studies are needed in this area.

### 3.8. Lipocalin-2

Lipocalin-2 is a recently discovered adipokine and a member of the lipocalin family. It has been speculated that lipocalin-2 is involved in insulin resistance in obesity [110]. While no recent studies have evaluated the dynamic of lipocalin-2 levels in physiological pregnancy specifically, much research has considered the relationship of circulating lipocalin-2 with pregnancy complications, as well as outcomes.

#### 3.8.1. Lipocalin-2 in GDM and PE

In a cohort study by Mierzyński et al., maternal serum lipocalin-2 concentrations at 24–29 weeks gestation were significantly higher in the GDM group when compared to those found in healthy pregnant controls [43]. They also observed a strong positive correlation between chemerin and lipocalin-2 levels in maternal serum in both the GDM group, as well as the control group, and suggested that both adipokines were involved in the pathophysiology of GDM [43]. A further prospective cohort study investigating lipocalin-2 levels at 11–13 weeks gestation and GDM found lipocalin-2 to be significantly higher in mothers who later developed GDM [111]. In a combined multivariate prediction model, lipocalin-2 in maternal serum, among other parameters, was found to be an independent predictor for early GDM [111].

Studies considering maternal lipocalin-2 levels and PE have also been published. A case-control study with 22 PE patients and 22 healthy age-matched controls observed lipocalin-2 levels in maternal serum to be significantly increased in PE patients in comparison to the control group [112]. A positive correlation was also found between maternal lipocalin-2 levels and parameters of PE, such as diastolic blood pressure and creatinine [112]. A second case-control study with 186 PE patients split into mild and severe PE and 72 healthy pregnant controls also found significantly elevated maternal lipocalin-2 levels in the PE group in comparison to healthy pregnant subjects [113]. Lipocalin-2 in maternal serum also correlated with the severity of PE, with higher lipocalin-2 concentrations in patients with severe PE when compared to mild PE patients. Among the three studied adipokines, which included serum soluble fms-like tyrosine kinase 1 (sFlt-1) and CXC chemokine ligand 16 (CXCCL16), lipocalin-2 was found to have the highest correlation with both diagnoses, as well as the severity of PE [113].

#### 3.8.2. Lipocalin-2 and Parameters of Intrauterine Growth

To examine the relationship between maternal circulating lipocalin-2 and parameters of intrauterine growth, Papasthanasiou et al., performed a cross-sectional study on a population including 80 mothers and their offspring, who were divided into AGA, LGA and IUGR groups [114]. They found a significant increase in maternal lipocalin-2 levels in mothers who later delivered AGA infants when compared to mothers who later delivered LGA neonates or neonates with IUGR [114].

#### 3.8.3. Cord Blood Lipocalin-2

In the previously mentioned cross-sectional study, lipocalin-2 in cord blood at birth was also analyzed [114]. Lipocalin-2 levels in cord blood were significantly increased when compared to levels found in maternal serum, and they were also significantly elevated in the IUGR group when compared to the LGA group [114].

Recent studies concerning lipocalin-2 suggest that lipocalin-2 is increased in maternal serum in both GDM and PE. Higher cord blood levels of lipocalin-2 are also associated with IUGR neonates.

### 3.9. Visfatin

Visfatin is a novel adipokine with direct associations with insulin secretion and diabetes. By binding to the insulin receptor at a site different from the binding site of insulin, it can interfere with glucose metabolism and lead to hypoglycemia [110]. Although it is secreted by several organs, it is highly expressed in visceral fat tissue.

#### 3.9.1. Visfatin in Physiological Pregnancy

Changes in maternal visfatin levels during physiological pregnancy have been examined in several studies. In a systematic review [15] considering adipokines and their role in GDM, de Gennaro et al., included the findings of a cohort study analyzing visfatin expression in normal healthy pregnancy [115]. Their data suggested an increase in visfatin expression in normal pregnancy [115]. It is speculated that this increase might lead to a higher nutritional supply of the fetus, allowing for optimal intrauterine growth [15,116].

#### 3.9.2. Visfatin in GDM and PE

Several studies considering visfatin levels in maternal serum during GDM were published in recent years. In a prospective cohort study, no differences in maternal visfatin levels were observed between GDM patients and healthy pregnant subjects [17]. Similar findings were also reported by five further studies, including two meta-analyses [16,20,101,117,118]. In their meta-analysis, including 24 studies and a total of 2305 participants, 1033 of which had GDM, Zhang et al., found that while maternal visfatin did not seem to correlate with GDM, it did correlate positively with maternal obesity, and suggested that it is therefore linked to GDM through maternal weight gain [118]. Nevertheless, a prospective cohort study found low maternal visfatin levels to be a risk factor for GDM [119], while another cohort study reported lower visfatin levels in obese women with GDM when compared to non-obese healthy controls [34]. Conversely, six studies reported an association between elevated maternal visfatin levels and impaired glucose tolerance during pregnancy. A prospective case-control study including 38 GDM patients and 35 age- and BMI-matched controls reported a positive correlation between maternal serum visfatin levels taken on the day of delivery and weight gain, as well as BMI in GDM patients [120]. They also found a negative correlation between serum visfatin and fasting plasma glucose [120]. These findings were supported by a cohort study, which reported higher visfatin levels in the serum of mothers with GDM in the second trimester in comparison to healthy pregnant subjects, and observed an association between maternal visfatin levels and serum ferritin, insulin, age and BMI [121]. Another cohort study also reported elevated maternal visfatin levels in GDM pregnant women and found an association between higher visfatin expression and a higher incidence of adverse outcomes [122]. Two case-control studies analyzing serum visfatin in the first trimester [24] as well as at delivery [103] also concluded that increased visfatin levels were associated with GDM. A systematic review further supported the predictive role of increased visfatin in GDM [62]. Another systematic review additionally suggested an association between maternal serum visfatin levels and the prediction of cardiovascular injuries related to GDM [106]. Furthermore, a cohort study evaluating maternal serum visfatin several years postpartum found no significant differences between the levels found in women with previous GDM and controls [123].

In PE, findings were more homogenous. Two studies reported similar maternal serum visfatin concentrations between PE patients and healthy pregnant controls [124,125]. In contrast, a cohort study found elevated serum visfatin levels in the third trimester of PE patients when compared with healthy pregnant controls and non-pregnant controls, and serum visfatin appeared to correlate with the severity of PE [126]. This trend was supported by two case-control studies [127,128], one of which reported elevated maternal visfatin levels in the first trimester in women who later developed PE in comparison to healthy controls [127]. Ferreira et al., also speculated that this increase was not associated with impaired placental perfusion [127]. Moreover, a strong linear correlation was found between serum visfatin in mothers with PE and systolic, diastolic and mean arterial blood pressure [128]. This association was also reported by a cross-sectional study [129]. Consequently, Zorba et al., proposed maternal serum visfatin to be a useful parameter in distinguishing preeclamptic from non-preeclamptic pregnancies [128]. The findings of a meta-analysis also supported increased maternal visfatin levels in PE pregnancies when compared to healthy controls [130]. Conversely, Chandrasekaran et al., concluded that while increased visfatin levels were observed in obese women with PE when compared to obese healthy controls, maternal serum visfatin levels did not differ significantly between obese and normal-weight patients with PE [32].

#### 3.9.3. Visfatin and Parameters of Intrauterine Growth

A case-control study evaluating maternal visfatin levels at delivery found a negative correlation between serum visfatin in women with GDM and birthweight as well as length at birth [103]. Similarly, in a cross-sectional study, elevated maternal visfatin levels were observed in women who delivered an SGA neonate when compared with normal pregnancies [124], while this trend was not observed between PE mothers with SGA offspring when compared to PE mothers without [124]. A systematic review also reported higher maternal visfatin levels in women who delivered preterm [131].

#### 3.9.4. Cord Blood Visfatin

In their case-control study, Mazaki-Tovi et al., reported lower visfatin concentrations in cord serum when compared to levels found in maternal serum [125]. They also found no significant differences in cord blood visfatin in term AGA offspring, SGA offspring or PE offspring when compared to gestational age-matched preterm AGA neonates [125]. The authors speculated that the source of elevated serum visfatin in mothers carrying an SGA neonate is not fetal circulation [125]. Another cohort study reported cord visfatin levels to be similar between SGA, AGA and LGA neonates, and proposed a negative association of cord visfatin with gestational weight gain [132]. However, a further cohort study found cord blood visfatin to be increased in infants with IUGR and LGA neonates when compared with AGA neonates, and this increase was higher in LGA infants [133]. After six months, visfatin levels decreased in LGA and IUGR offspring [133]. Visfatin levels in neonates were also found to be elevated in PE and GDM offspring [133].

In summary, although visfatin has been the subject of several studies in recent years, reports vary on the dynamic of maternal visfatin levels in GDM and PE. Higher maternal visfatin levels appear to be associated with SGA offspring and preterm birth. Further studies are needed in order to fully establish the role of visfatin in pregnancy pathologies.

### 3.10. NRG4

NRG4 has recently emerged as a novel adipokine and is a member of the neuregulin family. Although it is secreted by several organs, it is highly expressed in brown fat tissue, plays a central role in energy homeostasis, and is associated with an improvement of metabolic disorders such as insulin resistance [134].

#### NRG4 in GDM and PE

Only a few studies concerning circulating NRG4 in pregnancy have been published so far. In a case-control study, Kralisch et al., quantified NRG4 both during pregnancy and after delivery in 74 women with GDM and healthy pregnant controls, and compared postpartum levels in a follow-up study with 25 women who previously had GDM and 25 healthy control subjects. The median postpartum levels of NRG4 in maternal serum were similar to the levels found in prepartum [135]. The authors also reported lower serum levels of NRG4 in GDM women when compared with healthy pregnant controls [135]. On the other hand, a prospective cross-sectional study analyzing NRG4 levels at the 24th–28th gestational week in 63 GDM patients found higher serum NRG4 levels in GDM mothers compared to the levels found in the control group [136].

One case-control study investigated the association of maternal circulating NRG4 with PE. They found no significant differences between NRG levels in PE patients and healthy pregnant subjects, regardless of the severity of PE [137].

In summary, NRG4 is a newly emerging adipokine, and further studies are required to investigate its association with parameters of intrauterine growth and pregnancy outcomes. Conflicting reports exist concerning its role in pregnancy pathologies.

### 3.11. Progranulin

Progranulin, a glycoprotein, is a recently identified adipokine and is known to induce insulin resistance. It has been associated with inflammation, as well as wound repair and neurodegeneration [138]. Few studies concerning progranulin in pregnancy have been published.

#### 3.11.1. Progranulin in Physiological Pregnancy

In a cross-sectional cohort study, Ebert et al., concluded that progranulin levels in maternal serum are elevated during pregnancy, and this dysregulation is associated with pregnancy status and not with the presence of GDM [35].

#### 3.11.2. Progranulin in GDM and PE

The previous cross-sectional study did not find an association of maternal progranulin levels with the presence of GDM but rather an association with pregnancy status itself, suggesting that GDM does not lead to a change in progranulin levels in maternal serum [35]. However, another cross-sectional study evaluating PE patients and healthy pregnant women determined that mean progranulin levels in maternal serum were significantly higher in women with PE when compared with levels found in non-complicated pregnancies, and reported higher progranulin levels in women with early onset PE than those with late-onset PE [139]. They speculated that this elevation is a reflection of placental dysfunction associated with PE [139].

#### 3.11.3. Progranulin and Parameters of Intrauterine Growth

In the previous cross-sectional study, the authors also reported a negative correlation between maternal progranulin levels and the gestational age of neonates at birth, as well as birth weight [139].

Although few studies considering progranulin in pregnancy are available, they support an increase in maternal progranulin in PE and a negative correlation with birth weight. Further studies are required to investigate the association of cord progranulin with parameters of intrauterine growth and pregnancy outcome.

## 4. Discussion

Despite numerous publications on adipokines and their role in pregnancy in recent years, reports on their profiles during pregnancy and pregnancy pathologies, as well as their possible association with pregnancy outcomes, remain varied, as is demonstrated in Table 1, Table 2, Table 3 and Table 4.

### 4.1. Adipokines in Physiological Pregnancy

Few studies have analyzed adipokine levels in physiological gestation, yet in summary, they suggest a change in serum levels of various adipokines during pregnancy compared to the non-pregnant state. Whether these changes stem from placental co-secretion, from an increase of maternal adipose tissue volume, or from alterations of maternal metabolic processes, in general, remains unclear. What is known, however, is that metabolic processes change dramatically during pregnancy, which can lead to complications such as gestational diabetes, and that these complications during pregnancy are associated with an increased risk of metabolic disease later in life. The reviewed studies support the assumption that adipokines play an important role in the adaption of metabolic processes, such as changes in insulin sensitivity/resistance during physiological pregnancy. Nevertheless, the exact mechanisms underlying the changes in metabolic processes during gestation are still unknown. Furthermore, the direct relationship between adipokines in physiological pregnancy and various pregnancy parameters, such as gestational week and gestational weight gain, has also yet to be explained. Therefore, it is important to further understand the role of adipokines in the adaption of metabolic processes during (physiological) pregnancy and possibly establish standard values of adipokines in maternal serum that correspond with healthy gestation.

**Table 1:** Adipokines in physiological pregnancies.

Physiological Changes in Maternal Adipokine Levels during Pregnancy		
Resistin	↑	with advancing pregnancy [13] [12]
	↓	from first to second trimester [11]
Chemerin	↑	[35] [36] [37]
	↔	[13]
RBP4		-
GDF15	↑	[76] [77]
Irisin	↑	[83] ^1^
A FABP	↑	[15] ^2^
Omentin-1	↓	[98] [99]
Lipocalin-2		-
Visfatin	↑	[15]
NRG4	↔	[135] ^3^
Progranulin	↑	[35]

^1^ precursor of irisin elevated, ^2^ during hypoxic stress, ^3^ prepartum levels vs. postpartum levels.

### 4.2. Maternal Serum Adipokines in GDM

Reports on maternal serum levels of adipokines in GDM were generally scarce and yielded conflicting results for certain adipokines such as resistin, chemerin, RBP4, visfatin and NRG4 (Table 2). Results for other adipokines, such as AFABP and lipocalin-2 (increased in patients with GDM) or omentin-1 (decreased in patients with GDM), were more consistent. For some adipokines, only initial studies exist, suggesting a possible association with GDM, as is the case for GDF15 [78]. Additional studies are needed here on larger cohorts of GDM patients in order to refute or affirm these findings. Should these studies confirm the described associations between adipokines and GDM, and as the research surrounding adipokines in both physiological and complicated pregnancies expands, it might become possible in the future to identify certain adipokines as biomarkers for the prediction of gestational diabetes. Adipokines could, in turn, be used as markers of future metabolic risk in women after pregnancy, or even provide for new therapeutic approaches in treating insulin resistance and impaired glucose tolerance during gestation, therefore helping to prevent the slew of complications associated with GDM.

**Table 2:** Adipokines in GDM and PE.

Changes in Adipokine Levels in Maternal Serum during Pregnancy Pathologies				
	PE		GDM	
Resistin	↑	[12] [19] [24] [30] [31]	↑	[9] [22] [23] [24] [25]
	↔	[32] [33]	↔	[16] [17] [18] [19] [20] [21]
			↓	[27]
Chemerin	↑	[47] [48] [49] [50] [51] [52]	↑	[34] [36] ^1^ [41] [42] [43] [44] [45]
			↔	[13] [35] [38] [39] [40]
			↓	[36] ^2^ [37] [46]
RBP4	↑	[64] [65] [66] [67]	↑	[54] [55] [56] [57] [58]
	↔	[59] [68] [69] [70] [71]	↔	[34] [59] [60] [61] [62]
	↓	[63]		
GDF15	↑	[77] ^3^ [79] [80]	↑	[78]
	↔	[77] ^4^		
	↓	[76]		
Irisin			↔	[84] [85] ^5^
	↓	[83 ^1^	↓	[86]
A FABP	↑	[52] [92]	↑	[42] [46] [54] [62] [89] [90] [91]
			↔	[13] [26]
Omentin-1	↓	[107]	↓	[40] [99] [100] [101] [102] [103] [104] [105] ^6^
Lipocalin-2	↑	[112] [113]	↑	[43] [111]
Visfatin	↑	[32] ^7^ [126] [127] [128] [130]	↑	[24] [62] [103] [120] [121] [122]
	↔	[124] [125]	↔	[16] [17] [20] [101] [117] [118]
			↓	[34] ^7^ [119]
NRG4			↑	[136]
	↔	[137]		
			↓	[135]
Progranulin	↑	[139]		
			↔	[35]

^1^ In third trimester, ^2^ in first trimester, ^3^ in preterm PE, ^4^ non-preterm PE, ^5^ forms of DM other than GDM included, ^6^ in non-overweight vs. obese subjects, ^7^ in obese women.

### 4.3. Maternal Serum Adipokines in PE

Compared to GDM, study results for PE were more consistent, and various adipokines, including resistin, chemerin, AFABP, lipocalin-2, and visfatin, were described to be elevated in PE as compared to healthy pregnancies (Table 2). This strongly suggests that the aforementioned adipokines play a role in the pathophysiology of PE and possibly placental dysfunction. Furthermore, adipokines might also be associated with pro-inflammatory changes seen in PE and the increased risk for cardiovascular disease later on in life, which is seen in these patients. To this end, and with regard to PE, additional research is needed to further elucidate the role of adipokines in PE and its associated pathologies, as well as the potential of specific adipokines as biomarkers for the prediction of preeclampsia in women. Moreover, the possibility of using adipokines to better classify women’s risk of developing cardiovascular or metabolic disease later on in life remains to be evaluated.

### 4.4. Maternal Serum Adipokines and Parameters of Intrauterine Growth

Only a few studies investigating maternal adipokine levels and their association with parameters of intrauterine growth exist, and sample sizes are small, which makes it difficult to interpret the results (Table 3). In summary, little is known so far about the role of adipokines at the crossing point between intrauterine to postnatal life, if they play a role in fetal programming, whether they stem from the mother, the placenta or the fetus, and whether they are associated with postnatal metabolic adaptations. Although the literature supports the association of maternal adipokines with intrauterine growth, no information was available concerning the mechanisms that could possibly underlie this association. Additional studies on larger cohorts are again necessary in order to better understand these associations.

**Table 3:** Adipokines in and parameters of intrauterine growth.

Association of Maternal Serum Adipokine Levels with Parameters of Intrauterine Growth							
Resistin	Birthweight					↓	[30]
	LGA, SGA			↔	[19]		
Chemerin	Birthweight	↑	[41]			↓	[49]
	APGAR 1,5					↓	[49]
RBP4	Birthweight					↓	[72] ^1^
	FGR					↓	[68]
	SGA			↔	[59] [66]		
	LGA			↔	[59]		
	Preterm birth	↑	[65]				
GDF15		-					
Irisin	Higher birthweight			↔	[87] ^2^		
AFABP	Higher birthweight	↑	[93]				
Omentin-1	IUGR			↔	[108]		
	Preterm birth					↓	[100]
Lipocalin-2	AGA	↑	[114] ^3^				
Visfatin	Birthweight					↓	[103] ^4^
	Length at birth					↓	[103] ^4^
	SGA neonate	↑	[124]				
	Preterm birth	↑	[131]				
NRG4		-					
Progranulin	Birthweight					↓	[139]

^1^ only in SGA group, ^2^ Only in AGA group, ^3^ compared to IUGR, LGA ^4^ in GDM women.

### 4.5. Cord Blood Adipokines and Pregnancy Outcomes

Studies concerning cord blood adipokines and pregnancy outcomes were also limited, and therefore the validity of their findings on larger cohorts is again questionable (Table 4). Important questions which remain include whether or not cord blood adipokine levels correlate with birth weight as well as with growth during infancy. It also remains to be seen where cord blood adipokines originate from—are they mainly produced by the fetus, or are they transferred from the mother across the placenta, or are they instead produced in the placenta itself? The mechanism by which cord blood adipokines affect fetal growth and metabolism during pregnancy is yet undescribed; however, differences in cord adipokines between healthy offspring of physiological gestation and pathological pregnancy outcomes could implicate the potential role of adipokines in the fetal programming of metabolic processes. This might possibly give important new insight into the mechanisms which affect pre- and postnatal growth and metabolic processes and the increased risk for cardiovascular and metabolic disease later in life, which is described in children with impaired intrauterine growth and abnormally high or low birth weight.

**Table 4:** Adipokines in cord blood and pregnancy outcomes.

Association of cord Blood Adipokines with Various Pregnancy Outcomes				
Resistin	GDM offspring			↓ [27]
	higher birthweight	↑ [23]		
Chemerin	GDM offspring	↑ [38] ^1^		
	Obese mother	↑ [38] ^2^		
	Birthweight		↔ [38]	
RBP4	GDM offspring	↑ [73] ^3^		
	PE offspring			↓ [63]
	LGA	↑ [74]		
	Birthweight z score	↑ [74]		
GDF15	SGA		↔ [81]	
Irisin	GDM offspring		↔ [85]	
	Maternal BMI		↔ [85]	
	Neck circumference			↓ [87]
AFABP	GDM offspring	↑ [96] ^4^		
	hypoglycemic newborns	↑ [95]		
	SGA infants			↓ [94]
	Preterm birth	↑ [94]		
Omentin-1	GDM offspring			↓ [99]
	IUGR		↔ [108]	
	SGA	↑ [109]		
	LGA			↓ [109]
Lipocalin-2	IUGR	↑ [114] ^5^		
Visfatin	PE offspring	↑ [133]	↔ [125]	
	GDM offspring	↑ [133]		
	Term SGA, AGA		↔ [125]	
	LGA	↑ [133]	↔ [132]	
	IUGR	↑ [133]		
NRG4	-	-		
Progranulin	-	-		

^1^ in arterial cord blood, ^2^ in venous cord blood, ^3^ in females only, ^4^ in males only ^5^ compared to LGA.

### 4.6. Conclusions

In conclusion, findings suggest that adipokines may play an important role in the complex physiological changes that occur during pregnancy, and could have implications for the management of pregnancy-related health conditions. While the changes in adipokine levels in maternal serum could possibly be explained by placental co-secretion or as a result of the increase in maternal adipose tissue volume, further studies are needed to establish the role of adipokines in the metabolic changes present during physiological pregnancy. Although certain adipokines such as AFABP and lipocalin-2 seem to be increased in GDM, while omentin-1 is conversely decreased, the potential of using adipokines as biomarkers for GDM in pregnant women must also be intensively researched. Here arises the prospect of one day utilizing adipokines as a therapeutic approach to treat insulin resistance and its associated pathologies. Studies also suggested that adipokines such as chemerin and resistin were associated with PE and placental dysfunction. This leads to the question of whether adipokines in maternal serum could also serve as biomarkers for PE and help identify patients with a higher risk of cardiovascular and metabolic diseases in later life after PE. The reviewed literature also supported an association between adipokines both in maternal and cord blood with various parameters of intrauterine growth; however, further studies are required on larger cohorts in order to better understand this association. As knowledge in the field of adipokines and pregnancy increases, the focus is drawn to the possible role of adipokines as biomarkers of mother and child health. Ideally, this would allow for standard values of certain adipokines in maternal serum during gestation and in cord blood at birth to be established, paving the way for better prediction of pregnancy outcomes for both mother and child.

## Data Availability

Data sharing not applicable.

## Supplementary Materials

The following supporting information can be downloaded at: https://www.mdpi.com/article/10.3390/biomedicines11051419/s1, Table S1: Table of reviewed studies listed in order of appearance in the review article.
