# Supplementary material for: Adipokines in Pregnancy: A Systematic Review of Clinical Data
Source: Biomedicines. 2023 May 11;11(5):1419. doi: 10.3390/biomedicines11051419 (PMC10216846; doi:10.3390/biomedicines11051419)
Supplement: Supplementary file 1 [file biomedicines-11-01419-s001.zip › biomedicines-2348913-supplementary.pdf]

Reference Nr.	Author	Year	Paper Title	Study type
[11]	Al-Musharaf, S., et al.	2021	Inflammatory and Adipokine Status from Early to Midpregnancy in Arab Women and Its Associations with Gestational Diabetes Mellitus	Multi-center prospective study
[12]	Banjac, G., et al.	2021	The role of resistin in early preeclampsia prediction	Prospective cohort study
[13]	Guelfi, K., et al	2017	Maternal circulating adipokine profile and insulin resistance in women at high risk of developing gestational diabetes mellitus	Randomized controlled trial
[16]	Lobo, T., et al	2019	Adipokine levels in overweight women with early-onset gestational diabetes mellitus	Nested case-control study
[17]	Boyadzhieva, M., et al	2013	Adipocytokines during pregnancy and postpartum in women with gestational diabetes and healthy controls	Cohort study
[18]	Karatas, A., et al.	2014	Relationship of maternal serum resistin and visfatin levels with gestational diabetes mellitus	Case-control study
[19]	Nanda, S., et al.	2012	Maternal serum resistin at 11 to 13 weeks' gestation in normal and pathological pregnancies	Prospective cohort study
[20]	O'Malley, E., et al.	2020	The use of biomarkers at the end of the second trimester to predict Gestational Diabetes Mellitus	Prospective observational study
[21]	Lobo, T	2013	Resistin concentration and gestational diabetes: A systematic review of the literature	Meta-Analysis
[22]	Siddiqui, K., et al.	2018	Serum adipokines (adiponectin and resistin) correlation in developing gestational diabetes mellitus: pilot study	Case-control study
[23]	Shang, M., et al.	2018	Correlation of adipokines and markers of oxidative stress in women with gestational diabetes mellitus and their newborns	Prospective cohort study

[24]	Bawah, A., et al.	2019	Leptin, resistin and visfatin as useful predictors of gestational diabetes mellitus	Case-control study
[25]	Kapustin, R., et al.	2020	Maternal serum leptin, adiponectin, resistin and monocyte chemoattractant protein-1 levels in different types of diabetes mellitus.	Longitudinal prospective cohort study
[26]	Vorobjova, T., et al.	2022	FABP4 and I-FABP Levels in Pregnant Women Are Associated with Body Mass Index but Not Gestational Diabetes	Cohort study
[27]	McManus, R., et al	2014	Maternal, umbilical arterial and umbilical venous 25-hydroxyvitamin D and adipocytokine concentrations in pregnancies with and without gestational diabetes	Case-control study
[28]	Aviram, A., et al.	2020	The association between adipocytokines and glycemic control in women with gestational diabetes mellitus	Prospective observational trial
[29]	Seol, H., et al.	2010	Comparison of serum levels and the placental expression of resistin between patients with preeclampsia and normal pregnant women	Cohort study
[30]	Song, Y., et al.	2016	Serum levels of leptin, adiponectin and resistin in relation to clinical characteristics in normal pregnancy and preeclampsia	Case-control study
[31]	Talab, A., et al.	2021	Antepartum and postpartum changes in adipokines, endothelial dysfunction, inflammatory markers and other biochemical parameters in preeclamptic women: A prospective observational cohort study	Prospective observational cohort study
[32]	Chandrasekaran, S., et al	2020	Adipokine profiles in preeclampsia	Case-control study
[33]	Chandrasekaran, S., et al	2020	Evaluating Relationships between Visceral Fat Measures and Adipokines Concentrations among Women with a History of Preeclampsia	Secondary analysis of cross-sectional study
[34]	Tsiotra, P., et al.	2018	Circulating adipokines and mRNA expression in adipose tissue and the placenta in women with gestational diabetes mellitus	Cohort study

[35]	Ebert, T., et al.	2020	Adipocytokines are not associated with gestational diabetes mellitus but with pregnancy status	Cross-sectional cohort study
[36]	Yang, X.,	2017	Serum chemerin level during the first trimester of pregnancy and the risk of gestational diabetes mellitus	Cross-sectional study, cohort study
[37]	Hare, K., et al.	2014	Decreased plasma chemerin levels in women with gestational diabetes mellitus	Case-control study
[38]	Van Poppel, M., et al.	2014	Cord blood chemerin: Differential effects of gestational diabetes mellitus and maternal obesity	Observational, longitudinal study
[40]	Sun, J., et al.	2020	Circulating apelin, chemerin and omentin levels in patients with gestational diabetes mellitus: A systematic review and meta-Analysis	Meta-analysis
[39]	Zhou, Z., et al.	2018	Circulating chemerin levels and gestational diabetes mellitus: A systematic review and meta-analysis	Meta-analysis
[41]	Fatima, S., et al.	2017	Elevated levels of chemerin, leptin, and interleukin-18 in gestational diabetes mellitus	Cohort study
[42]	Francis, E., et al.	2020	Adipokines in early and mid-pregnancy and subsequent risk of gestational diabetes: A longitudinal study in a multiracial cohort	Longitudinal cohort study
[43]	Mierzyński, R., et al.	2021	The Potential Role of Chemerin, Lipocalin 2, and Apelin in the Diagnosis and Pathophysiology of Gestational Diabetes Mellitus	Cohort study
[44]	Li, X., et al.	2015	Chemerin expression in Chinese pregnant women with and without gestational diabetes mellitus	Cohort study
[45]	Schuitemaker, J., et al.	2020	First trimester secreted Frizzled-Related Protein 4 and other adipokine serum concentrations in women developing gestational diabetes mellitus	Nested case-control study

[46]	Mosavat, M., et al.	2021	Adipocyte-Specific Fatty Acid-Binding Protein (AFABP) and Chemerin in Association with Gestational Diabetes: A Case-Control Study	Case-control study
[47]	Stepan, H., et al.	2011	Serum levels of the adipokine chemerin are increased in preeclampsia during and 6months after pregnancy	Case-control study
[48]	Duan, D., et al.	2012	Serum levels of the adipokine chemerin in preeclampsia	Case-control study
[49]	Cetin, O., et al.	2017	Chemerin level in pregnancies complicated by preeclampsia and its relation with disease severity and neonatal outcomes	Cohort study
[50]	Chen, Y., et al.	2023	Third-Trimester Maternal Serum Chemerin and Hypertension After Preeclampsia: A Prospective Cohort Study.	Prospective cohort study
[51]	Xu, Q., et al.	2014	The predictive value of the first-trimester maternal serum chemerin level for pre-eclampsia	Prospective cross-sectional study
[54]	Zhang, Y., et al	2016	Changes in serum adipocyte fatty acid-binding protein in women with gestational diabetes mellitus and normal pregnant women during mid- and late pregnancy	Prospective cohort study
[55]	Abetew, D., et al.	2013	Association of retinol binding protein 4 with risk of gestational diabetes	Cohort study
[56]	Du, C., et al.	2019	A prospective study of maternal plasma concentrations of retinol-binding protein 4 and risk of gestational diabetes mellitus	Prospective observational study
[57]	Hu, S., et al.	2016	Serum level and polymorphisms of retinol-binding protein-4 and risk for gestational diabetes mellitus: A meta-analysis	Meta-analysis
[58]	Huang, Q., et al.	2015	Circulating retinol-binding protein 4 levels in gestational diabetes mellitus: A meta-analysis of observational studies	Meta-analysis
[59]	Nanda, S., et al.	2013	Maternal serum retinol-binding protein-4 at 11-13 weeks' gestation in normal and pathological pregnancies	Cohort study

[60]	Jia, X., et al.	2022	The relationship between serum adipokine fibroblast growth factor-21 and gestational diabetes mellitus	Cohort study
[61]	Gursoy, A., et al.	2015	Early second trimester retinol-binding protein-4 values in cases with or without gestational diabetes mellitus risk factors: A cross-sectional study	Cross-sectional study
[63]	Seol, H., et al.	2011	Retinol-binding protein-4 is decreased in patients with preeclampsia in comparison with normal pregnant women.	Case-control study
[64]	Yliniemi, A., et al.	2015	First trimester placental retinol-binding protein 4 (RBP4) and pregnancy-associated placental protein A (PAPP-A) in the prediction of early-onset severe pre-eclampsia	Case-control study
[65]	Mendola, O., et al.	2017	Retinol-binding protein 4 and lipids prospectively measured during early to mid-pregnancy in relation to preeclampsia and preterm birth risk	Observational longitudinal study
[66]	Vaisbuch, E., et al.	2010	Retinol binding protein 4 - A novel association with early-onset preeclampsia	Cross-sectional study
[67]	Hamdan, H., et al.	2020	Association between Retinol-Binding Protein 4 Levels and Preeclampsia: A Systematic Review and Meta-Analysis	Meta-analysis
[68]	Fruscalzo, A., et al	2013	Retinol binding protein as early marker of fetal growth restriction in first trimester maternal serum	case-control study
[69]	Zhang, Y., et al.	2018	Association between plasma retinol binding protein 4 levels and severe preeclampsia	Case-control study
[70]	Palalioglu, R., et al.	2023	Evaluation of maternal serum SERPINC1, E-selectin, P-selectin, RBP4 and PP13 levels in pregnancies complicated with preeclampsia	Prospective cohort study
[71]	Masuyama, H., et al.	2011	Retinol-binding protein 4 and insulin resistance in preeclampsia	Case-control study

[72]	Fruscalzo, A., et al.	2017	First trimester TTR-RBP4-ROH complex and angiogenic factors in the prediction of small for gestational age infant's outcome	Case-control study
[73]	Yang, M., et al.	2020	Sex dimorphism in the associations of gestational diabetes with cord blood adiponectin and retinol-binding protein 4	Nested case-control study
[74]	Yang, M., et al.	2020	Retinol-binding protein 4, fetal overgrowth and fetal growth	Case-control study
[76]	Chen, Q., et al.	2016	Serum levels of GDF15 are reduced in preeclampsia and the reduction is more profound in late-onset than early-onset cases	Case-control study
[77]	Wertaschnigg, D., et al.	2020	Second- and third-trimester serum levels of growth-differentiation factor-15 in prediction of pre-eclampsia	Case-control study
[78]	Yakut, K., et al.	2021	Is GDF-15 level associated with gestational diabetes mellitus and adverse perinatal outcomes?	Case-control study
[79]	Cruickshank, T., et al.	2021	Circulating growth differentiation factor 15 is increased preceding preeclampsia diagnosis: Implications as a disease biomarker	Prospective cohort study
[80]	Yuksel, I., et al.	2018	Maternal levels of growth differentiation factor-15 in patients with preeclampsia	Cross-sectional study
[81]	Diaz, M., et al.	2020	Circulating growth-and-differentiation factor-15 in early life: relation to prenatal and postnatal growth and adiposity measurements	Cohort study
[83]	Garcés, M., et al.	2014	Irisin levels during pregnancy and changes associated with the development of preeclampsia	Prospective cohort study
[84]	Ebert, T., et al.	2014	Serum levels of irisin in gestational diabetes mellitus during pregnancy and after delivery	Case-control study
[85]	Hernandez-Trejo, M., et al.	2016	Relationship between irisin concentration and serum cytokines in mother and newborn	Cohort study

[86]	Kuzmicki, M., et al.	2014	Serum irisin concentration in women with gestational diabetes	Case-control study
[87]	Ökdemir, D., et al.	2018	The Role of Irisin, Insulin and Leptin in Maternal and Fetal Interaction	Cohort study
[91]	Sun, J., et al.	2020	Circulating FABP4, nesfatin-1, and osteocalcin concentrations in women with gestational diabetes mellitus: A meta-analysis	Meta-analysis
[92]	Lin, Y., et al.	2018	Study on the correlation between adipocyte fatty-acid binding protein, glucolipid metabolism, and pre-eclampsia	Cohort study
[93]	Ciborowski, M., et al.	2014	Potential first trimester metabolomic biomarkers of abnormal birth weight in healthy pregnancies	Cross-sectional study
[94]	Joung, K., et al.	2017	Cord Blood Adipocyte Fatty Acid–Binding Protein Levels Correlate With Gestational Age and Birth Weight in Neonates	Cross-sectional study
[95]	Ron, I., et al.	2021	The adipokine FABP4 is a key regulator of neonatal glucose homeostasis	Cohort study
[96]	Liu, X., et al.	2021	Sex Dimorphic Associations of Gestational Diabetes Mellitus With Cord Plasma Fatty Acid Binding Protein 4 and Estradiol	Nested case-control study
[98]	Aktas, G., et al.	2014	Association between omentin levels and insulin resistance in pregnancy	Cohort study
[99]	Franz, M., et al.	2018	Maternal and neonatal omentin-1 levels in gestational diabetes	Prospective case-control study
[100]	Mierzyński, R., et al.	2018	Adiponectin and omentin levels as predictive biomarkers of preterm birth in patients with gestational diabetes mellitus	Cohort study
[101]	Abell, S., et al.	2017	The association between dysregulated adipocytokines in early pregnancy and development of gestational diabetes	Randomized trial

[102]	Abell, S., et al.	2019	Role of serum biomarkers to optimise a validated clinical risk prediction tool for gestational diabetes	Randomized trial
[103]	Souvannavong-Vilivong, X., et al	2019	Placental expressions and serum levels of adiponectin, visfatin, and omentin in GDM	Prospective cohort study
[104]	Pan, X., et al.	2019	Omentin-1 in diabetes mellitus: A systematic review and meta-analysis	Meta-analysis
[105]	Papatheodorou, S., et al.	2022	Association between omentin-1 and indices of glucose metabolism in early pregnancy: a pilot study	Cohort study
[107]	Liu, H., et al.	2015	Association of serum omentin-1 concentrations with the presence and severity of preeclampsia	Cross-sectional study
[108]	Briana, D., et al.	2011	Omentin-1 and vaspin are present in the fetus and neonate, and perinatal concentrations are similar in normal and growth-restricted pregnancies	Case-control study
[109]	Hernández-Rodríguez, C., et al.	2019	Differences in omentin-1 levels in term newborns according to birth weight	Secondary analysis, cohort study
[111]	Sweeting, A. et al.	2019	A Novel Early Pregnancy Risk Prediction Model for Gestational Diabetes Mellitus	Prospective cohort study
[112]	Stepan, H., et al.	2010	Serum levels of the adipokine lipocalin-2 are increased in preeclampsia	Case-control study
[113]	Li, L., et al.	2021	Expression and significance of serum soluble fms-like tyrosine kinase 1 (sFlt-1), CXC chemokine ligand 16 (CXCL16), and lipocalin 2 (LCN-2) in pregnant women with preeclampsia	Case-control study
[114]	Papasthanasiou, A., et al.	2021	Perinatal lipocalin-2 profile at the extremes of fetal growth	Cross-sectional study
[117]	Jiang, Y., et al.	2021	Visfatin level and gestational diabetes mellitus: a systematic review and meta-analysis,	Meta-analysis

[118]	Zhang, W., et al.	2018	Association between circulating visfatin and gestational diabetes mellitus: a systematic review and meta-analysis	Meta-analysis
[119]	Park, S., et al.	2013	Gestational diabetes is associated with high energy and saturated fat intakes and with low plasma visfatin and adiponectin levels independent of prepregnancy BMI	Cohort study
[120]	Liang, Z., et al.	2016	Correlations of serum visfatin and metabolisms of glucose and lipid in women with gestational diabetes mellitus	Prospective case-control study
[121]	Kaygusuz, I., et al.	2013	Serum levels of visfatin and possible interaction with iron parameters in gestational diabetes mellitus	Cohort study
[122]	Lorenzo-Almorós, A., et al.	2019	A clinical research study on the respective relationships between visfatin and human fetuin A and pregnancy outcomes in gestational diabetes mellitus	Cohort study
[123]	Molęda, I., et al.	2016	Adipokines and β-cell dysfunction in normoglycemic women with previous gestational diabetes mellitus	Case-control study
[124]	Mazaki-Tovi, S., et al.	2010	Could alterations in maternal plasma visfatin concentration participate in the phenotype definition of preeclampsia and SGA?	Cross-sectional study
[125]	Mazaki-Tovi, S., et al.	2010	Maternal and neonatal circulating visfatin concentrations in patients with pre-eclampsia and a small-for-gestational age neonate	Case-control study
[126]	Zulfikaroglu, E., et al.	2010	Plasma visfatin levels in preeclamptic and normal pregnancies	Cohort study
[127]	Ferreira, A., et al.	2013	Maternal serum visfatin at 11-13 weeks' gestation in preeclampsia	Case-control study
[128]	Zorba, E., et al.	2012	Visfatin serum levels are increased in women with preeclampsia: A case-control study	Case-control study

[129]	Shaheen, A., et al.	2022	Relationship of serum visfatin levels with serum electrolytes, liver profile, hepatic enzymes and anthropometric parameters in pregnant women with preeclampsia and eclampsia during 3rd trimester	Cross-sectional study
[130]	Amiri-Dashatan, N., et al.	2020	Association between circulating visfatin and pre-eclampsia: a systematic review and meta-analysis	Meta-analysis
[132]	Estrada-Zúñiga, C., et al.	2019	Are cord blood visfatin concentrations different depending on birth weight category?,	Cohort study
[133]	Shemi, M., et al.	2016	Effect of intrauterine growth pattern on serum visfatin concentrations in full-term infants at birth and at 6 months of life	Prospective cohort study
[135]	Kralisch, S., et al.	2018	The brown-fat-secreted adipokine neuregulin 4 is decreased in gestational diabetes mellitus	Case-control study
[136]	Kurek Eken M., et al.	2018	Clinical significance of neuregulin 4 (NRG4) in gestational diabetes mellitus	Cross-sectional study
[137]	Yakut, K., et al.	2022	Comparison of maternal serum NRG-4 levels in healthy and preeclamptic pregnancies	Cohort study
[139]	Açikgöz, A., et al.	2016	Evaluation of maternal serum progranulin levels in normotensive pregnancies, and pregnancies with early- and late-onset preeclampsia	Cross-sectional study
